# Let-7i-5p represses brite adipocyte function in mice and humans

**DOI:** 10.1038/srep28613

**Published:** 2016-06-27

**Authors:** Maude Giroud, Michael Karbiener, Didier F. Pisani, Rayane A. Ghandour, Guillaume E. Beranger, Tarja Niemi, Markku Taittonen, Pirjo Nuutila, Kirsi A. Virtanen, Dominique Langin, Marcel Scheideler, Ez-Zoubir Amri

**Affiliations:** 1 Univ. Nice Sophia Antipolis, iBV, UMR 7277, Nice, 06100, France; 2 CNRS, iBV UMR 7277, 06100 Nice, 06100, France; 3 Inserm, iBV, U1091, 06100 Nice, 06100, France; 4 Department of Phoniatrics, ENT University Hospital, Medical University, Graz, Austria; 5 Department of Surgery, Turku University Hospital, Turku, 20521, Finland; 6 Department of Anesthesiology, Turku University Hospital, Turku, 20521, Finland; 7 Turku PET Centre, University of Turku, Turku, 20521, Finland; 8 Department of Endocrinology, Turku University Hospital, Turku, 20521, Finland; 9 Inserm, UMR1048, Obesity Research Laboratory, Institute of Metabolic and Cardiovascular Diseases, Toulouse, France; 10 University of Toulouse, UMR1048, Paul Sabatier University, Toulouse, France; 11 Toulouse University Hospitals, Department of Clinical Biochemistry, Toulouse, France; 12 Institute for Diabetes and Cancer (IDC), Helmholtz Zentrum München, German Research Center for Environmental Health, Neuherberg, Germany; 13 Joint Heidelberg-IDC Translational Diabetes Program, Heidelberg University Hospital, Heidelberg, Germany; 14 Molecular Metabolic Control, Medical Faculty, Technical University Munich, Germany; 15 German Center for Diabetes Research (DZD), Neuherberg, Germany

## Abstract

In response to cold or β3-adrenoreceptor stimulation brown adipose tissue (BAT) promotes non-shivering thermogenesis, leading to energy dissipation. BAT has long been thought to be absent or scarce in adult humans. The recent discovery of thermogenic brite/beige adipocytes has opened the way to development of novel innovative strategies to combat overweight/obesity and associated diseases. Thus it is of great interest to identify regulatory factors that govern the brite adipogenic program. Here, we carried out global microRNA (miRNA) expression profiling on human adipocytes to identify miRNAs that are regulated upon the conversion from white to brite adipocytes. Among the miRNAs that were differentially expressed, we found that Let-7i-5p was down regulated in brite adipocytes. A detailed analysis of the Let-7i-5p levels showed an inverse expression of UCP1 in murine and human brite adipocytes both *in vivo* and *in vitro*. Functional studies with Let-7i-5p mimic in human brite adipocytes *in vitro* revealed a decrease in the expression of UCP1 and in the oxygen consumption rate. Moreover, the Let-7i-5p mimic when injected into murine sub-cutaneous white adipose tissue inhibited partially β3-adrenergic activation of the browning process. These results suggest that the miRNAs Let-7i-5p participates in the recruitment and the function of brite adipocytes.

Obesity has reached epidemic proportions worldwide, with more than 1.9 billion overweight adults and approximately 600 million of them being obese^1^. Obesity constitutes a substantial risk factor for hypertension, type 2 diabetes, and cardiovascular diseases, which put a tremendous burden on public health care^2^,^3^. A positive energy balance, *i.e.* increased energy intake vs. energy expenditure, leads to an increase in body weight mainly due to an increase in the adipose tissue mass. The adipose organ can be divided into two distinct types of adipose tissues, white and brown. White adipose tissue (WAT) is specialized in the storage and release of fat^4^. In contrast, brown adipose tissue (BAT) dissipates energy by producing heat (thermogenesis) via the uncoupling of the activity of the mitochondrial electron transport chain through the specific expression of uncoupling protein 1 (UCP1)^5^,^6^. BAT is composed of brown adipocytes characterized by a high mitochondrial content and endowed with a high capacity of lipid oxidation^7^. In addition to the thermogenic brown adipocytes located in BAT, WAT contains thermogenic fat cells, called “brown-in-white” (“brite”) or “beige” adipocytes, which are able to burn fat and carbohydrates via non-shivering thermogenesis^8^,^9^. Brite adipocytes appear as islands formed upon chronic PPARγ activation, β3-adrenergic or cold stimulation, but their precise origin remains controversial. Recently, *in vivo* lineage studies clearly showed that brite adipocytes can be derived from white adipocytes upon cold exposure and can trans-differentiate into white (whitening) adipocytes upon thermoneutrality exposure^10^,^11^,^12^. However, other studies showed that a proportion of brite adipocytes appearing in subcutaneous WAT (scWAT) upon cold exposure originate from *de novo*-differentiated adipocytes^13^. It is now well admitted, that BAT constitutes a target to combat obesity and associated diseases^14^. One therapeutic option is to convert and activate a proportion of white into brite adipocytes, which may lead to normalization of metabolic parameters and consequently to body weight loss. Several regulatory determinants have already been reported to play key roles in adipose tissue function among which microRNAs (miRNAs) have emerged as a novel class of endogenous modulators of adipose tissue function. Importantly, miRNAs constitute potential “druggable” targets that are involved in the switch between mature white and brite adipocytes^15^,^16^. miRNAs are small (~22 nt long) non-coding RNAs that regulate gene expression post-transcriptionally by specifically binding to the 3′ untranslated region of target mRNAs to adjust protein output either by mRNA destabilization or by translational repression^17^,^18^,^19^. Their impact on diseases is acknowledged by their development as drugs and/or drug targets in phase 1 and 2 clinical trials^20^. Interestingly, miRNAs have also been shown to be involved in human adipocyte differentiation, lipid metabolism, diabetes and obesity^21^,^22^,^23^,^24^,^25^,^26^. Recently, the miR-193b-365 cluster has been described as a novel regulator of closely related brown and myogenic lineages. In addition, miR-196a and miR30b/c have been described as the first non-coding RNA that acts as a key regulator of brite adipocyte development in mice and humans^24^,^27^,^28^,^29^,^30^. Very recently, miRNAs have been shown to play dual roles, by simultaneously repressing cytoplasmic targets and activating mitochondrial mRNAs, highlighting the importance of these regulators in the control of mitochondriogenesis that takes place during white to brite adipocyte conversion^31^. However, the involvement of miRNAs in white to brite/brown adipocyte conversion has not been reported so far.

Herein, we performed a global analysis using hMADS cells to identify miRNAs that were important for browning of white adipocytes. We identified Let-7i-5p as a potential candidate. Together, our observations showed that Let-7i-5p may play a crucial role in the modulation of brite adipocytes function, and thus, close regulation of its levels might help to shift human adipocytes from an energy storing to an energy dissipating fate.

## Results

### Global profiling identified differentially expressed miRNAs during conversion of white adipocytes to brite adipocytes

**Figure 1:**
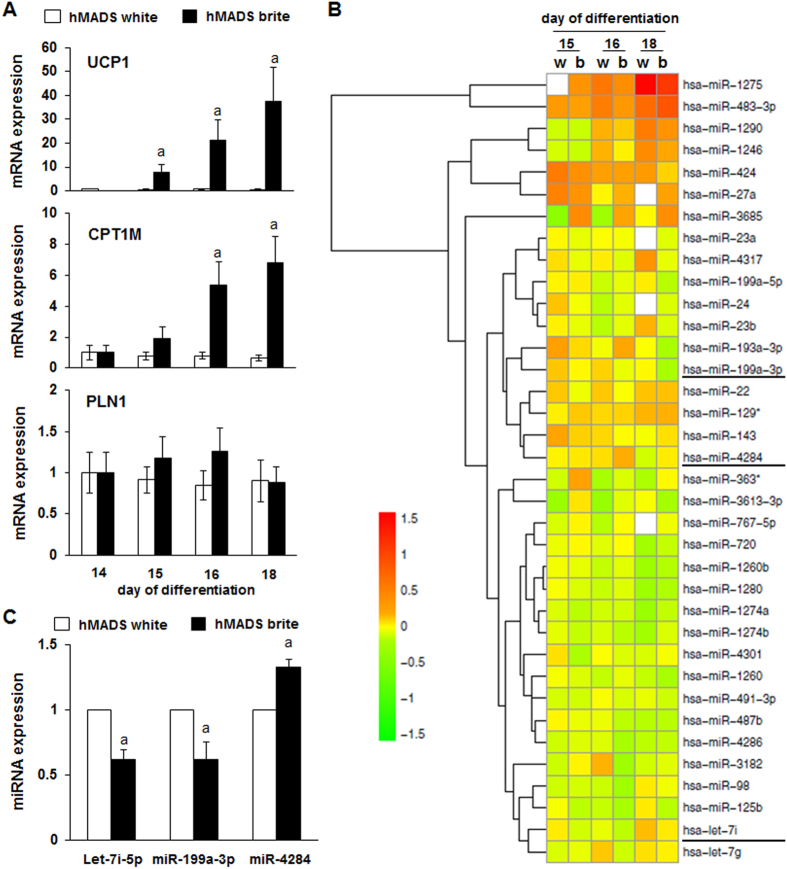
Global analysis of miRNAs regulated during browning of hMADS cells. hMADS cells were differentiated into white or brite adipocytes upon rosiglitazone treatment between days 14 and 18 as described in the materials and methods section. (**A**) UCP1, CPT1M and PLN1 mRNA expression relative to the white adipocyte condition determined by RT-qPCR. (**B**) Heat map of miRNA microarray performed on hMADS cells treated or not with rosiglitazone at day 15, 16 or 18. White fields indicate missing values due to stringent data filtering. (**C**) RT-qPCR validation of various miRNA at day 18. Results are mean +/− SEM of 4 (**A**) and 6 (**C**) independent experiments performed on different series of cells. a: p < 0.05.

We performed global miRNA profiling on human adipocytes to identify miRNAs and pathways important for browning of white adipocytes. Human multipotent adipose-derived stem (hMADS) cells were cultured under adipogenic conditions. After differentiation into white adipocytes (day 14), a PPARγ agonist (100 nM rosiglitazone) was added to the medium for 1, 2 or 4 days to induce a brite adipocyte phenotype. Cells were analyzed for the expression of UCP1, CPT1M and perilipin (PLN1) mRNA and subsequently subjected to global miRNA expression profiling. As expected, PLN1 expression, a marker of adipogenesis, was not affected, in contrast to UCP1 and CPT1M mRNA levels that increased gradually depending on the duration of the rosiglitazone treatment (Fig. 1A).

Microarray data analysis yielded a list of miRNAs that were regulated by PPARγ agonist treatment at days 15, 16 and 18 of differentiation (Fig. 1B). Our data showed that the differential miRNA expression was predominantly subtle and that approximately 33% of the miRNAs were up- and 33% were down-regulated, whereas 33% were not regulated (data not shown). We validated by RT-qPCR analysis the down-regulation of Let-7i-5p and miR-199a-3p and the up-regulation of miR-4284 during the conversion of white to brite adipocytes (Fig. 1C). Thus, our data showed that the conversion of hMADS adipocytes induced the regulation of various miRNAs including Let-7i-5p. As other members of the Let-7 miRNA family have been reported to modulate the UCP1 level in bone marrow derived adipocytes^32^, we chose to focus our study on Let-7i-5p for further characterization.

### Let-7i-5p levels were lower in brite than white adipocytes in humans

**Figure 2:**
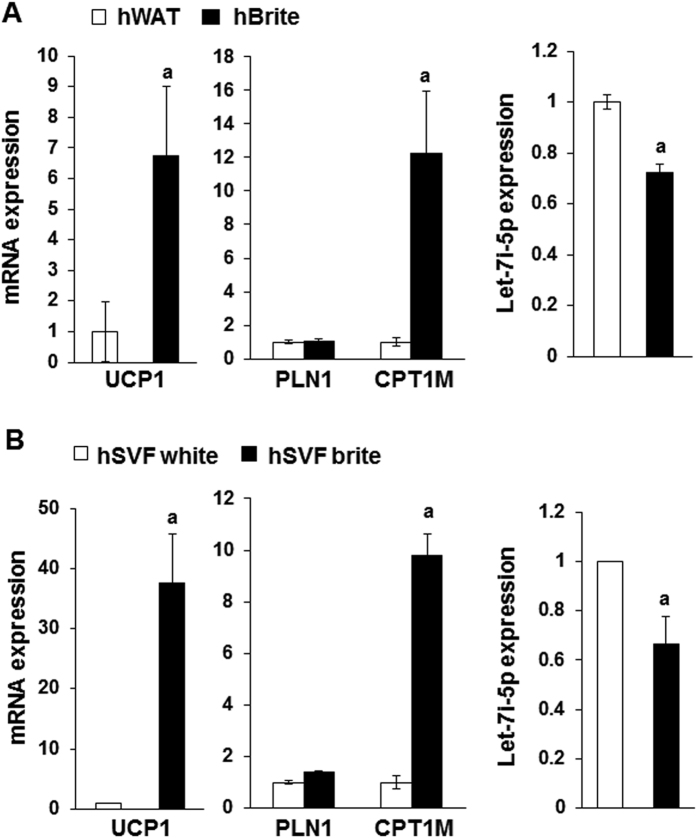
Let-7i-5p levels in human adipose tissue and cell models. (**A**) mRNA and miRNA levels were evaluated by RT-qPCR in matched biopsies from 7 healthy human adult patients of adipose depots negative and positive for FDG incorporation. (**B**) mRNA and miRNA expression determined in SVF-derived white and brite adipocytes obtained from 3 human subcutaneous adipose tissue samples. Results are mean +/− SEM. a: p < 0.05.

In addition to the analysis performed in hMADS cells during white to brite adipocyte conversion, we decided to investigate Let-7i-5p expression in experimental settings that were relevant to human physiology. First, we quantified Let-7i-5p in human supraclavicular adipose tissue biopsies from cold exposed healthy volunteers and isolated after FDG-PET/CT analysis. Positive and negative samples for glucose uptake were sampled and correspond respectively to WAT containing (named hBrite) or not (named hWAT) brite adipocytes. As expected, the expression of UCP1 and CPT1M mRNA was higher in hBrite samples compared to hWAT samples whereas expression of PLN1 mRNA was equivalent in both samples. Notably and in line with our previous *in vitro* results, the levels of Let-7i-5p were lower in hBrite samples compared to hWAT (Fig. 2A).

To further corroborate these findings, we used another *in vitro* model, *i.e*. cells of the stroma vascular fraction (SVF) from three human subcutaneous adipose tissue donors that were induced to differentiate into white or brite adipocytes. Again, UCP1 and CPT1M mRNA expression was confined to brite adipocytes, whereas the Let-7i-5p level was lower in brite compared to white adipocytes (Fig. 2B). PLN1 expression, used as a marker of the level of adipogenesis, was similar under these conditions (Fig. 2B).

### Let-7i-5p over-expression inhibited UCP1 mRNA expression and oxygen consumption in human brite adipocytes

**Figure 3:**
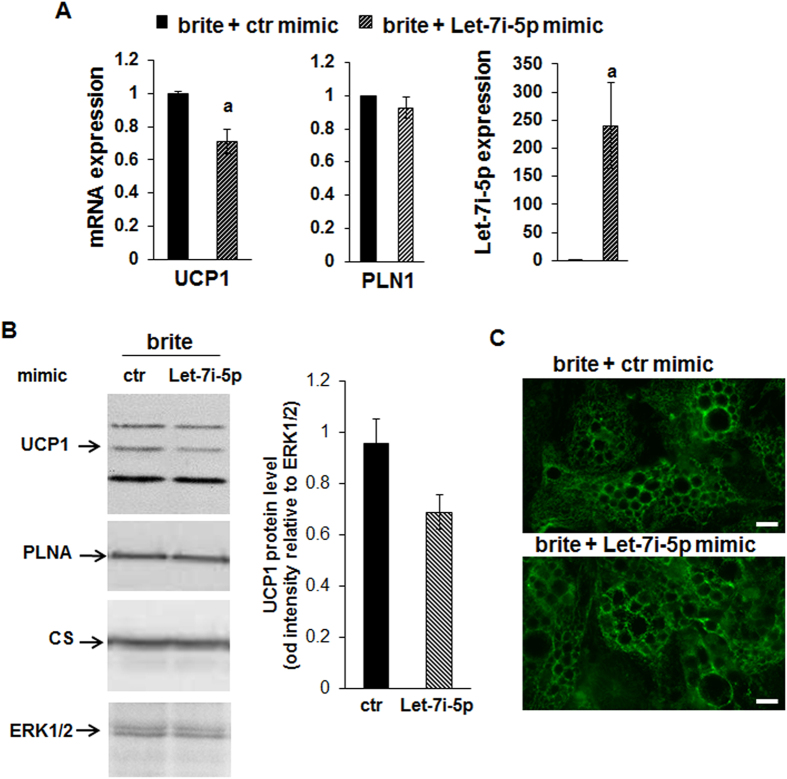
Effects of Let-7i-5p over-expression on hMADS brite adipocytes. hMADS cells were transfected with a Let-7i-5p mimic or a control mimic at day 14 and then differentiated into brite adipocyte and analyzed at day 18. (**A**) mRNA and miRNA expression was evaluated by RT-qPCR. (**B**) UCP1 and PLNA protein levels were evaluated by immunoblotting. ERK1/2 was used as a loading control. Histograms show the quantification of the band intensities (n = 2). (**C**) Immunostaining for UCP1. Results are mean +/− SEM of 6 independent experiments performed on different series of cells. a: p < 0.05. Scale bar: 20 μm.

**Figure 4:**
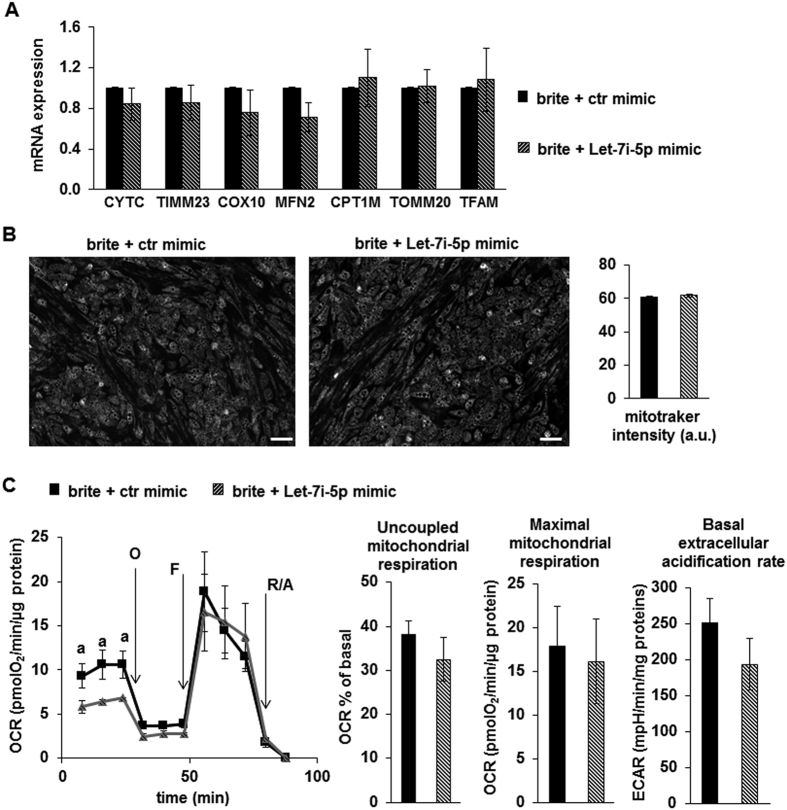
Effects of Let-7i-5p over-expression on the mitochondria function of hMADS brite adipocytes. hMADS white adipocytes were transfected with the Let-7i-5p mimic or control mimic at day 14 and then induced into brite adipocytes and analyzed at day 18. (**A**) mRNA expression of various key mitochondrial markers evaluated by RT-qPCR. (**B**) Alive adipocytes were incubated with MitoTraker Green as a probe for mitochondria independently of their activities and the signal was quantified and is presented as histograms. (**C**) Oxygen consumption measurements were performed at day 18. Plots show cellular OCR (O: oligomycin; F: FCCP; R/A: rotenone/antimycin **A**), and histograms show mitochondrial respiration values as well as the ECAR assessed under basal condition. Plots and histograms show the mean ± SEM, n = 6 (**A** & **B**) or 8 (**C**). a: p < 0.05. Scale bar: 150 μm.

We asked whether Let-7i-5p modulated the human brite adipocyte phenotype. Therefore, hMADS adipocytes were transfected with Let-7i-5p or control (ctr) mimics before induction of white to brite adipocyte conversion. The efficiency of transfection was substantial as shown by the high level of Let-7i-5p in transfected adipocytes (Fig. 3A). We observed a significant decrease in the UCP1 mRNA and protein levels in transfected brite adipocytes (Fig. 3A,B and Supplementary Fig. 1A). Under these conditions adipogenesis was not affected as the levels of PLN1 mRNA and protein (Fig. 3A,B) as well as other adipogenic markers were not affected (Supplementary Fig. 1B). However, the increase in Let-7i-5p expression did not affect the addressing of UCP1 to the mitochondria as shown on immuno-staining microphotographs (Fig. 3C). To define the impact of a decrease in Let-7i-5p-induced UCP1 expression, we analyzed various parameters of mitochondria. The citrate synthase (CS) protein level was not affected by transfection with the Let-7i-5p mimic (Fig. 3B), as for the mRNA levels of other classical mitochondria markers, including brite adipocyte specific markers such as CPT1M, (Fig. 4A and Supplementary Fig. 1B). Let-7i-5p over-expression did not affect mitochondriogenesis as the mitochondrial content, evaluated with a fluorescent mitochondrial probe, was similar under both conditions (Fig. 4B).

In association with UCP1 mRNA expression, hMADS brite adipocytes displayed a higher level of cellular respiration compared to white adipocytes^33^,^34^. We thus analyzed the oxygen consumption rate in Let-7i-5p transfected hMADS brite adipocytes. Interestingly, we observed that over-expression of Let-7i-5p led to a significant decrease in the basal oxygen consumption rate without affecting uncoupled respiration or the maximal respiration rate (Fig. 4C). Of note, this decreased basal respiration was not related to a glycolytic shift as the extracellular pH, indicative of lactate secretion, was not affected (Fig. 4C). In conclusion, transfection of the Let-7i-5p mimic inhibited partially UCP1 expression, which resulted in a decrease in the basal mitochondria activity, but not sufficiently to impact on the uncoupling activity.

### Let-7i-5p levels were low in mice brite adipocytes

**Figure 5:**
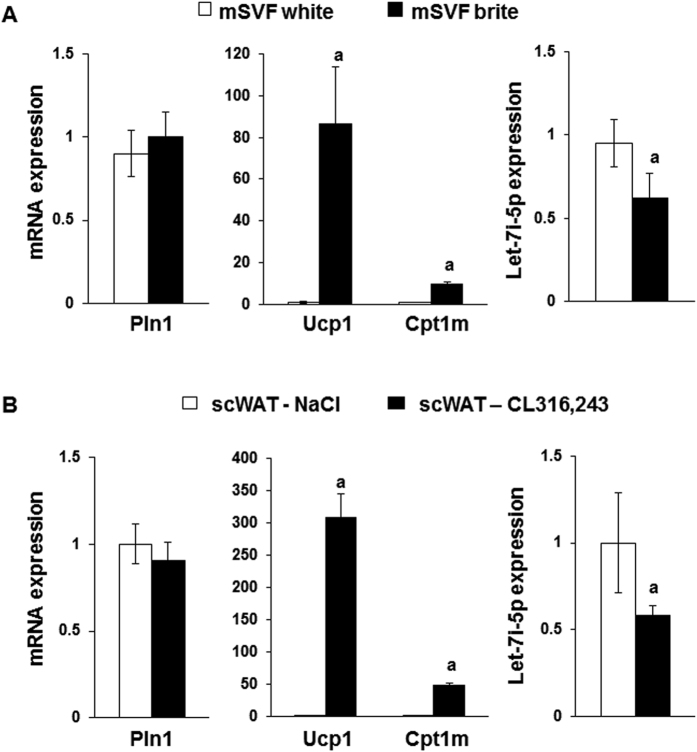
Let-7i-5p levels in mouse adipose tissue and cell models. (**A**) Cells from SVF of scWAT were differentiated into white or brite adipocytes and used for mRNA and miRNA level quantification by RT-qPCR. (**B**) mRNA and miRNA expression determined by RT-qPCR in scWAT from C57BL/6 mice treated or not with CL316,243 for 1 week. Control animals were injected with vehicle (NaCl 0.9%, w/v). Histograms represent mean ± SEM of 3 independent experiments (**A**) of 8 mice (**B**). a: p < 0.05.

SVF cells from murine scWAT were induced to differentiate either into white or brite adipocytes. As expected, the Ucp1 and Cpt1m mRNA were barely detected in *in vitro*-derived white adipocytes and were highly expressed in brite adipocytes (Fig. 5A). In agreement with data from human cells, Let-7i-5p levels were significantly lower in brite adipocytes compared to white-*in vitro*-derived adipocytes (Fig. 5A).

To determine whether a correlation exists between miRNA expression and brite adipocytes *in vivo*, mice, maintained at thermoneutrality (28–30 °C), received β3-adrenergic receptor agonist treatment (CL316,243, 1 mg/kg/day) for 1 week. This stimulation induced a significant increase in Ucp1 and Cpt1m mRNA expression in scWAT, which was associated with recruitment and activation of brite adipocytes (Fig. 5B). In striking similarity to our *in vitro* data, let-7i-5p levels in mice scWAT decreased with CL316,243 treatment (Fig. 5B). Altogether, these observations led us to assume that Let-7i-5p was a negative regulator of brite adipocyte formation and function.

### Let-7i-5p over-expression in murine scWAT limited recruitment of brite adipocytes

**Figure 6:**
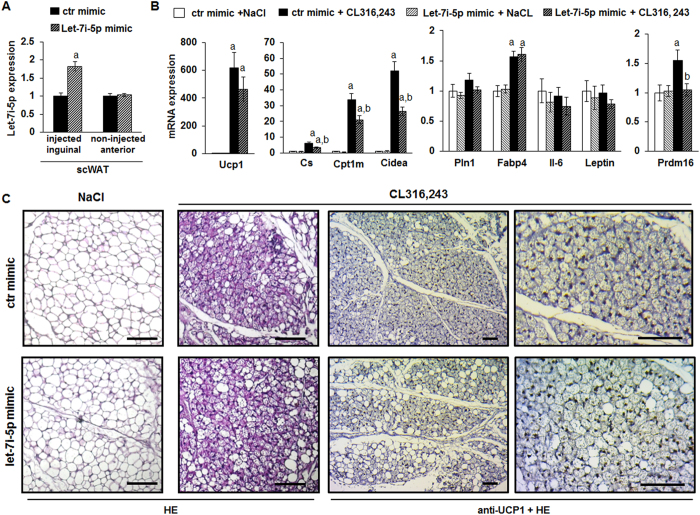
Effects of Let-7i-5 p injection into scWAT. 10 week-old C57BL/6 male mice received the Let-7i-5p or control mimics in the scWAT as described in the materials and methods section. Mice were subdivided into two groups, one group was treated with CL316,243 and the other group with vehicle (NaCl). (**A**) The Let-7i-5p level was evaluated in inguinal (mimic injected) and anterior (non-injected) scWAT 2 days after surgery. (**B**) Ucp1 and representative white and brite adipocyte mRNA markers were evaluated by RT-qPCR. (**C**) Representative histological sections of scWAT after the different treatments (7 μm, paraffin-embedded), as indicated HE staining (HE) or UCP1 immunostaining counterstained with HE (anti-UCP1 + HE) are shown. Histograms represent mean ± SEM of 4 (**A**) or 12 (**B**) mice. a: p < 0.05. a, b: p < 0.05. a: NaCl *vs.* CL316,243; b: ctr mimic *vs.* Let-7i-5p mimic. Scale bar: 100 μm.

We aimed at investigating *in vivo* whether let-7i-5p modulates recruitment and activation of brite adipocytes. For this, we injected let-7i-5p mimics directly into the scWAT of C57BL/6 mice. As the injections were restricted to the inguinal fat pads, we expect only local changes and no systemic modification. In line with this, body weight was similar in the different groups of mice (Supplementary Fig. 2A). 48 hours after injection, the scWAT of injected mice showed a higher level of Let-7i-5p demonstrating the efficacy of the injection (Fig. 6A). To investigate the effects of the Let-7i-5p mimic on brite adipocyte formation and activation in adipose tissues, mice received injections of β3-adrenergic receptor agonist CL316,243 (1 mg/kg/day) or vehicle one week after surgery for 7 days. Molecular analysis of the scWAT showed in Let-7i-5p compared to control mimic-injected mice an impaired increase in brite adipocytes and mRNA expression of mitochondrial markers such as Ucp1, CS, Cpt1m and Cidea with CL316,243 treatment (Fig. 6B). However, no effect on the Pln1, Fabp4, Il6 and leptin mRNA levels was observed (Fig. 6B). Interestingly, the increased expression of Prdm16 mRNA due to β3-adrenergic receptor agonist stimulation was blunted by Let-7i-5p mimic injection (Fig. 6B). These results were specific to the injected fat pad as the anterior scWAT, from the same mice that was not-injected with the mimics, did not display any inhibition of brite adipocyte marker expression after CL316,243 injection (Supplementary Fig. 2B). Moreover, histological analysis and UCP1 immunostaining showed a slight defect in the number of brite adipocytes (UCP1 positive and multilocular lipid droplet-containing adipocytes) upon let-7i mimic injection (Fig. 6C and Supplementary Fig. 2C). Taken together, these observations corroborated on a decrease in recruitment and/or activation of brite adipocytes, upon β3-adrenergic receptor agonist stimulation, due to Let-7i-5p over-expression.

## Discussion

Increasing evidences indicates that miRNAs play an important role in adipogenesis and in the control of brown adipocyte formation and function and as such represent promising therapeutic targets. Only a few miRNAs have been reported to play a crucial role in the formation and function of brown adipocytes, including the miR-26 family, miR-27, miR-133, miR-155, miR-196a and the clusters miR-106b-93 andmiR-193b-365^15^,^16^,^24^. In the present study we found that Let-7i-5p was negatively associated with UCP1 expression in brite compared to white adipocytes in murine and human tissues and cell models. We showed that Let-7i-5p affected brite adipocyte function *in vitro* through the specific inhibition of UCP1 expression, which impaired the mitochondrial oxygen consumption. The effect of murine let-7i-5p *in vivo* was more robust as its injection into scWAT impaired the formation and function of brite adipocytes.

Let-7 is one of the first to be described members of the large class of non-protein-coding RNAs. The Let-7 family is composed of 13 miRNA precursors giving 10 distinct mature Let-7 miRNAs, which are highly conserved, across species, in sequence and function^35^. Dysregulated expression of the Let-7 family is associated with various diseases such as cancer, cardiovascular and lung disease^36^. Furthermore, the Let-7 family is reported to be involved in the regulation of glucose metabolism and the control of insulin sensitivity as well as in the control of adipocyte differentiation^37^,^38^,^39^. Our data showed that Let-7i-5p levels were lower both in human biopsies of brite adipocytes compared to those of white adipocytes and in human primary culture-derived brite adipocytes. In agreement with this observation, the Let-7i-5p levels in murine scWAT dropped when the browning process was favored, *e.g.* upon β3-adrenergic stimulation. Of note, *in vitro* differentiation of scWAT precursors into white or brite adipocytes led to lower levels of Let-7i-5p in brite adipocytes. Altogether, these data point to an impact of Let-7i-5p levels on brite adipocyte formation and function in both mice and humans. Over-expression of Let-7i-5p led to inhibition of the basal oxygen consumption and decreased UCP1 expression in hMADS brite adipocytes. Using miRNA-target prediction tools, we found that UCP1 was not a putative target of Let-7i-5p. It is tempting to postulate that Let-7i-5p might control brite adipocyte function and formation through the modulation of transcriptional factors or co-factors that lie upstream of the brite adipocyte activation pathway. *In vivo*, direct injection of the Let-7i-5p mimic into the scWAT strongly inhibited β3-adrenergically-induced “browning” gene expression, including Ucp1, Prdm16, citrate synthase, Cpt1m and Cidea. Further investigations, such as the identification of target genes, will shed further light on the role of Let-7i-5p in the development of brite adipocytes. Indeed, *in silico* analysis showed that Let-7i-5p was able to target genes involved in mitochondriogenesis and mitochondrial function.

The other miRNAs identified in our microarray could be of great interest. For example, miR-4284, identified only in humans and not yet associated to any function in adipocytes, have as predicted targets *in silico* the well-known obesity associated gene FTO. Moreover, FTO has very recently been implicated in browning of human adipocytes^40^.

In conclusion, we described that Let-7i-5p levels were associated with brite adipocyte formation and function in mice and humans and may represent a potential new regulator of energy expenditure.

## Materials and Methods

### Reagents

Cell culture media and buffers were purchased from Lonza (Ozyme, St-Quentin en Yvelines, France), fetal bovine serum, insulin and trypsin from Invitrogen (Cergy Pontoise, France), and other reagents from Sigma-Aldrich Chimie (Saint-Quentin Fallavier, France).

### Animals

Experiments were conducted in accordance with French regulations for the care and use of research animals and were approved by the local experimentation committee (Nice University and Ciepal Azur: protocol NCE-2013-166). Animals were maintained under constant temperature (28 ± 2 °C) and 12:12-hour light-dark cycles, with *ad libitum* access to standard chow diet and water. 10 week-old male C57Bl/6J wild-type mice were from Janvier Labs. Brite adipocyte recruitment and activation was induced by intra-peritoneal injection of β-adrenergic receptor agonist, CL316,243 (1 mg/kg/day in saline solution) during 7 days. Control mice were injected with vehicle only.

For injection of miRNA mimics, mice were anesthetized with a Xylasin/Ketamine mixture. miRIDIAN Let-7i-5p and control mimics were delivered using *Invivo*-jetPEI reagent (Ozyme) according to manufacturer’s recommendations. *In vivo*-jetPEI does not induce any significant inflammatory response. Intra-subcutaneous WAT administration was carried out after making a longitudinal incision in the skin at the inguinal area. Mice received five injections per inguinal fat pad. Each injection contained 40 ng mimic Let-7i-5p in 10 μl. Mice were stitched and reanimated under warm light. Control mice groups were injected with mimic control. β-adrenergic receptor stimulation was carried out the last week by daily intra-peritoneal injections of CL316,243 (1 mg/kg in saline solution). Mice were sacrificed for analysis 2 days or 2 weeks after injection of mimics.

### Subjects

The study protocol was reviewed and approved by the ethics committee of the Hospital District of Southwestern Finland, and subjects provided written informed consent following the committee’s instructions. The study was conducted according to the principles of the Declaration of Helsinki. All potential subjects were screened for their metabolic status, and only those with normal glucose tolerance and a normal cardiovascular status (as assessed on the basis of electrocardiograms and measured blood pressure) were included. The age range of the subjects was 23–49 years. We studied a group of seven healthy volunteers (2 men and 5 women). Supraclavicular WAT containing brite adipocytes was sampled at a positive FDG-PET scan site, and subcutaneous WAT was derived via the same incision.

## Cell culture

### hMADS cell culture

The establishment and characterization of hMADS cells have been previously described^41^,^42^. Cells were used between passages 14 and 25, and all experiments were performed at least 3 times. Cells were seeded at a density of 5000 cells/cm^2^ in Dulbecco’s modified Eagle’s medium (DMEM) supplemented with 10% FBS, 15 mM Hepes, 2.5 ng/ml hFGF2, 60 mg/ml penicillin, and 50 mg/ml streptomycin. hFGF2 was removed when cells reached confluence. Cells were induced to differentiate at day 2 post-confluence (designated as day 0) in DMEM/Ham’s F12 media supplemented with 10 μg/ml transferrin, 10 nM insulin, 0.2 nM triiodothyronine, 1 μm dexamethasone and 500 μm isobutyl-methylxanthine (IBMX). Two days later, the medium was changed (dexamethasone and IBMX omitted) and 100 nM rosiglitazone were added. At day 9 rosiglitazone was withdrawn to enable white adipocyte differentiation but again included between days 14 and 18 to promote white to brite adipocyte conversion as we previously described^33^,^43^,^44^. Media were changed every other day and cells were used for analyses at day 18.

miRNA transfection assays were performed at day 14 with 5 nM of mimic and HiPerfect (Qiagen), following a previously described procedure^25^. miRIDIAN mimic control (ctr) and mimic Let-7i-5p were from Thermo Fischer Scientific.

### Mouse adipose tissue SVF preparation and culture

Animals were euthanized by cervical dislocation for isolation of stromal vascular fractions (SVF) as described previously^45^,^46^. Briefly, subcutaneous fat depots were rapidly excised, washed in phosphate-buffered saline, and minced. Adipose tissue samples were then digested for 45 min at 37 °C in DMEM containing 2 mg/ml collagenase A (Roche Diagnostics) and 20 mg/ml bovine serum albumin (Sigma Aldrich) with mild agitation. The lysate was successively filtrated through 250, 100 and 27 μm nylon sheets, and centrifuged for 5 min at 500 g. The pellet containing SVF cells was submitted to a red blood cell lysis procedure. SVF cells were plated and maintained in DMEM containing 10% FCS until confluence. Differentiation was induced in the same medium supplemented with 1 μm dexamethasone, 0.5 mM IBMX and 860 nM insulin. Dexamethasone and IBMX were omitted 2 days later and cells were maintained for 7–10 days in the presence of 20 nM insulin for white adipogenesis and 20 nM insulin, 1 μm rosiglitazone and 2 nM triiodothyronine for brite adipogenesis. Media were changed every other day.

### Human adipose tissue SVF preparation and culture

Abdominal sub-cutaneous human adipose tissue was collected from healthy patients as *res nullus* from surgeries (non-pathologic abdominoplasty) for SVF isolation according to the procedure described for mice (see above). SVF cells were either used directly for molecular analysis or plated and maintained in DMEM containing 10% FCS until confluence. Differentiation of primary cultures was performed according to the protocol described for hMADS cells.

### Isolation and analysis of RNA

These procedures followed MIQE standard recommendations and were conducted as described previously^43^. Total RNA was extracted using a TRI-Reagent kit according to the manufacturer’s instructions (Invitrogen). For analysis of mRNA from organs, tissues were homogenized in TRI-Reagent using an ULTRA TURRAX T25 (Ika, Germany). Two micrograms of total RNA, digested with Dnase I (Promega), were subjected to reverse transcription-polymerase chain reaction (RT-qPCR) analysis as described previously^43^. The oligonucleotide sequences, designed using Primer Express software, are shown in Supplementary Table 1.

For miRNA analysis, 10 ng of total RNA was reverse transcribed using Universal cDNA synthesis kit (Exiqon). qPCR and data evaluation were performed as described previously^25^,^47^. U6 and 5S were used as endogenous controls. The primer sequences used are described in Supplementary Table 1.

### MicroRNA microarray hybridization and data analysis

The miRNA screen was performed using miRCURY LNA™ microRNA Array Probe Set from Exiqon, to identify miRNA regulated during the “britening” of the hMADS adipocytes^48^. For hybridization, 200 ng total RNA extracts from 4 biological replicates of hMADS cells differentiated at the indicated days was hybridized against a common reference pool RNA (day 9) from all samples. End-labeling of miRNAs was performed using the Exiqon Power Labeling Kit (Exiqon, Denmark) together with a synthetic spike-in controls according to the manufacturer’s instructions. Slides were hybridized over night at 56 °C in a Tecan HS 400 hybridization station, followed by automated washing and drying with nitrogen (Tecan, Austria). Immediately after drying, arrays were scanned using the GenePix 4000B microarray scanner (Axon Instruments) at 10 μM resolution, followed by pre-processing to filter out low intensity, saturated and in homogenous spots. Background correction and normalization (global mean followed by dye-swap pair normalization) were performed, using ArrayNorm software^49^. The resulting log_2_-fold changes of miRNAs (calculated against the common reference sample) were further analysed and visualized using R (www.r-project.org). Normalized as well as raw microarray data are available upon request.

### Histological analysis

Histological analysis was performed as previously described^44^. Briefly, dissected adipose tissues were fixed overnight in formaldehyde and then paraffin-embedded. 7 μm adipose tissue sections were dewaxed. Immunohistochemistry for UCP1 was then performed following the manufacturer’s instructions (LSAB + system-HRP, Dako, Les Ulis, France) and using goat anti-UCP1 (clone C-17, Santa Cruz, Tebu-bio, Le Perray-en-Yvelines, France). Finally, sections were counterstained with haematoxylin-eosin (HE) and mounted with Entellan^®^ (Merck Chemical, Darmstadt, Germany).

### Immunoblot analysis

Cells were lysed in TNET lysis buffer (25 mM Tris-Cl (pH 7.4), 100 mM NaCl, 1mM EDTA, 1% Triton X-100, 0.5% Nonidet P40, 1× protease inhibitor cocktail and 1× Phosphostop mix (Roche Diagnostics, Meylan, France)). The protein concentration was evaluated with the BCA assay according to manufacturer’s recommendations (PIERCE, Thermo Scientific, France). Proteins were blotted using a basic SDS-PAGE protocol. Primary antibody incubation was performed overnight at 4 °C (anti-UCP1, Calbiochem #662045, dilution 1:750; anti-perilipin, Interchim #BP5015, dilution 1:5000; anti-ERK1/2, Cell Signaling #9102, 1:2000; anti-CS, Abcam #ab96600, dilution 1:10000). Primary antibodies were detected with HRP-conjugated anti-rabbit or anti-mouse immunoglobulins (Promega) and anti-guinea pig immunoglobulins (Santa Cruz). Detection was performed using Immobilon Western Chemiluminescent HRP Substrate (Merk-Millipore). OD band intensities were evaluated using PCBas Software.

### Immunostaining analysis

Cells were fixed with PAF 4% for 10 min, permeabilized with 0.1% Triton X-100 for 10 min, and then sequentially incubated with primary antibody overnight at 4 °C in a humidified chamber (anti-UCP1, Calbiochem, #662045, dilution 1:100) and with secondary antibody coupled with Alexa 488 (Invitrogen, dilution 1:500) for 30 min at room temperature. A PBS wash was performed twice between all steps. Cells were finally mounted in Mowiol and visualized with an Axiovert microscope (Carl Zeiss, Le Pecq, France) under oil immersion and pictures were captured and treated with AxioVision software (Carl Zeiss).

For mitochondria quantification, cells were incubated with MitoTracker® Green FM (Invitrogen) following the manufacturer’s protocol. Cells were washed and visualized with an Axiovert microscope and the fluorescence intensity evaluated using ImageJ.

### Measurement of metabolic parameters

For respiration analysis, hMADS cells were seeded in 24 multi-well plates and differentiated as previously described^34^,^50^. At day 14 of differentiation, hMADS cells were transfected with mimic Let-7i or mimic control and converted in brite adipocytes. Oxygen consumption rate (OCR) and extracellular acidification rate (ECAR) of 18 day-old differentiated cells was determined using an XF24 Extracellular Flux Analyzer (Seahorse Bioscience). Uncoupled and maximal OCR was determined using oligomycin (1.2 μm) and FCCP (1 μm). Rotenone (1 μm) and Antimycin A (1 μm) were used to inhibit Complex I- and Complex III-dependent respiration, respectively. Parameters were measured for each well using different value as previously described^51^.

### Statistical analyses

Data are expressed as mean values ± SEM and were analyzed using InStat software (GraphPad Software, CA, USA). Data were analyzed by Student’s *t*-test or one-way ANOVA followed by a Student-Newman-Keuls post-test. Differences were considered statistically significant when p < 0.05.

## Additional Information

**How to cite this article**: Giroud, M. *et al*. Let-7i-5p represses brite adipocyte function in mice and humans. *Sci. Rep.* **6**, 28613; doi: 10.1038/srep28613 (2016).

## Supplementary Material

Supplementary Information
