# Supplementary material for: Let-7i-5p represses brite adipocyte function in mice and humans
Source: Sci Rep. 2016 Jun 27;6:28613. doi: 10.1038/srep28613 (PMC4921928; doi:10.1038/srep28613)

Let-7i-5p represses brite adipocyte function in mice and humans

Maude Giroud[1,2,3], Michael Karbiener[4], Didier F. Pisani[1,2,3], Rayane A. Ghandour[1,2,3], Guillaume E. Beranger[1,2,3], Tarja Niemi[5], Markku Taittonen[6], Pirjo Nuutila[7,8], Kirsi A. Virtanen[7], Dominique Langin[9,10,11], Marcel Scheideler[12,13,14,15] and Ez-Zoubir Amri[1,2,3*]

Supplemental Table 1: Sequences used for mRNA and miRNA analysis.

**Human oligonucleotide sequences**		
**name**	**Reverse primer**	**Forward primer**
h36B4	TGCATCAGTACCCCATTCTATCAT	AGGCAGATGGATCAGCCAAGA
hCOX10	CACACTCTCTCCTCACGCCTC	TTCTTTCAAGATACCAGACAGAGC
hCPT1M	GAGCAGCACCCCAATCAC	AACTCCATAGCCATCATCTGCT
hCYTC	TGGCCCCTCCCATCTACAC	ATCCTTGGCTATCTGGGACATG
hPLN1	ACCCCCCTGAAAAGATTGCTT	GATGGGAACGCTGATGCTGTT
hTFAM	AATATGGTGCTGAGGAGTGTTAAAAG	TAACTGGTTTCCTGTGCCTATCC
hTIMM23	TGCATTTGGTGTCATCATTGAG	GGTTCCAGCTGCTACTGTGTTAAG
hTOMM20	GGGTTCTGTTGGGCATCTTTAA	AAAAAATGCAGGGCCTGGTT
hPPARγ1	TCGAGGACACCGGAGAGG	TGTGGTTTAGTGTTGGCTTCTTTC
hPPARγ2	CAAACCCCTATTCCATGCTGTT	ATCAGTGAAGGAATCGCTTTCTG
hFABP3	AGTTGGGGGTGGAGTTCGATGAGAC	GCAGTGCCGTGGGTGAGTGTC
hFABP4	TGTGCAGAAATGGGATGGAAA	CAACGTCCCTTGGCTTATGCT
hPGC1α	CTGTGTCACCACCCAAATCCTTAT	TGTGTCGAGAAAAGGACCTTGA
hGPDH	CCAGCGAGGTGGCTGATG	GTGTCTGCATCAGCTCTTTC
hADPQ	GCAGTCTGTGGTTCTGATTCCATAC	GCCCTTGAGTCGTGGTTTCC
hPRDM16	GAAACTTTATTGCCAATAGTGAGATGA	CCGTCCACGATCTGCATGT
hMFN2	TCCCTGCTAGGAGTTGCTGTAC	CACCTCAGCCCATGTGTCTCTT
hCIDEA	GCGAGAGTCACCTTCGACTTG	CGTTAAGGCAGCCGATGAA
hPDK4	TGGCAAGCCGTAACCAAAAC	ATGGATAATTCCCGGAATGCT
hUCP1	CCAGGATCCAAGTCGCAAGA	GTGTGCCCAACTGTGCAATG
**Murine oligonucleotide sequences**		
**name**	**Reverse primer**	**Forward primer**
m36B4	TCCAGGCTTTGGGCATCA	CTTTATCAGCTGCACATCACTCAGA
mCidea	CTGTCTCAATGTCAAAGCCACG	GAACTGTCCCGTCATCTGTGC
mCpt1m	GGCTCCAGGGTTCAGAAAGT	TGCCTTTACATCGTCTCCAA

mCs	AGCCCTCAACAGTGAAAGCAA	CTTGGCAATGAGGTCCAT
mFabp4	CTTGTGGAAGTCACGCCTTT	AAGAGAAAACGAGATGGTGACAA
mIl-6	CAAGAAAGACAAAGCCAGAGTCCT	TTATGTAAATCTTTTACCTCTTGGTTGAA
mLeptin	GCTGGTGAGGACCTGTTGAT	CAGGATCAATGACATTTCACACA
mPln1	AGCGTGGAGAGTAAGGATGTC	CTTCTGGAAGCACTCACAGG
mUcp1	CACCTTCCCGCTGGACACT	CCTGGCCTTCACCTTGGAT
**miRNA oligonucleotide sequences**		
**name**	**oligonucleotide sequence target**	
hsa-Let-7i-5p	UGAGGUAGUAGUUUGUGCUGUU	
hsa-miR-199a-3p	ACAGUAGUCUGCACAUUGGUUA	
hsa-miR-4284	GGGCUCACAUCACCCCAU	

Supplemental Figure 1. Effect of Let-7i-5p over-expression on hMADS brite adipocytes, additional data. hMADS cells were transfected with the Let-7i-5p mimic or control mimic at day 14 and then differentiated into brite adipocytes and analyzed at day 18. A) Uncropped immunoblot for UCP1 and corresponding ERK1/2 used as loading control. B) mRNA expression evaluated by RT-qPCR. Results are mean +/- SEM of 6 independent experiments performed on different series of cells.

Supplemental Figure 2. Effect of Let-7i-5p injection into scWAT, additional data. 10 week-old C57BL/6 male mice received the Let-7i-5p or control mimics into subcutaneous WAT as described in the materials and methods section. A) Mean weight of mice (12 by group) at different time points of the experiment. B) Ucp1 and representative white and brite adipocyte marker mRNA were determined by RT-qPCR in anterior scWAT from mice treated with CL316,243 or with vehicle (NaCl). Plots and histograms represent mean ± SEM of 12 mice. a: $p<0.05$, NaCL *vs.* CL316,243. C) Supplemental histological views of scWAT after different treatments are shown in addition to Figure 6. Hematoxylin and eosin staining (HE) and immunostaining for UCP1 protein (anti-UCP1 + HE).

Supplemental Figure 1

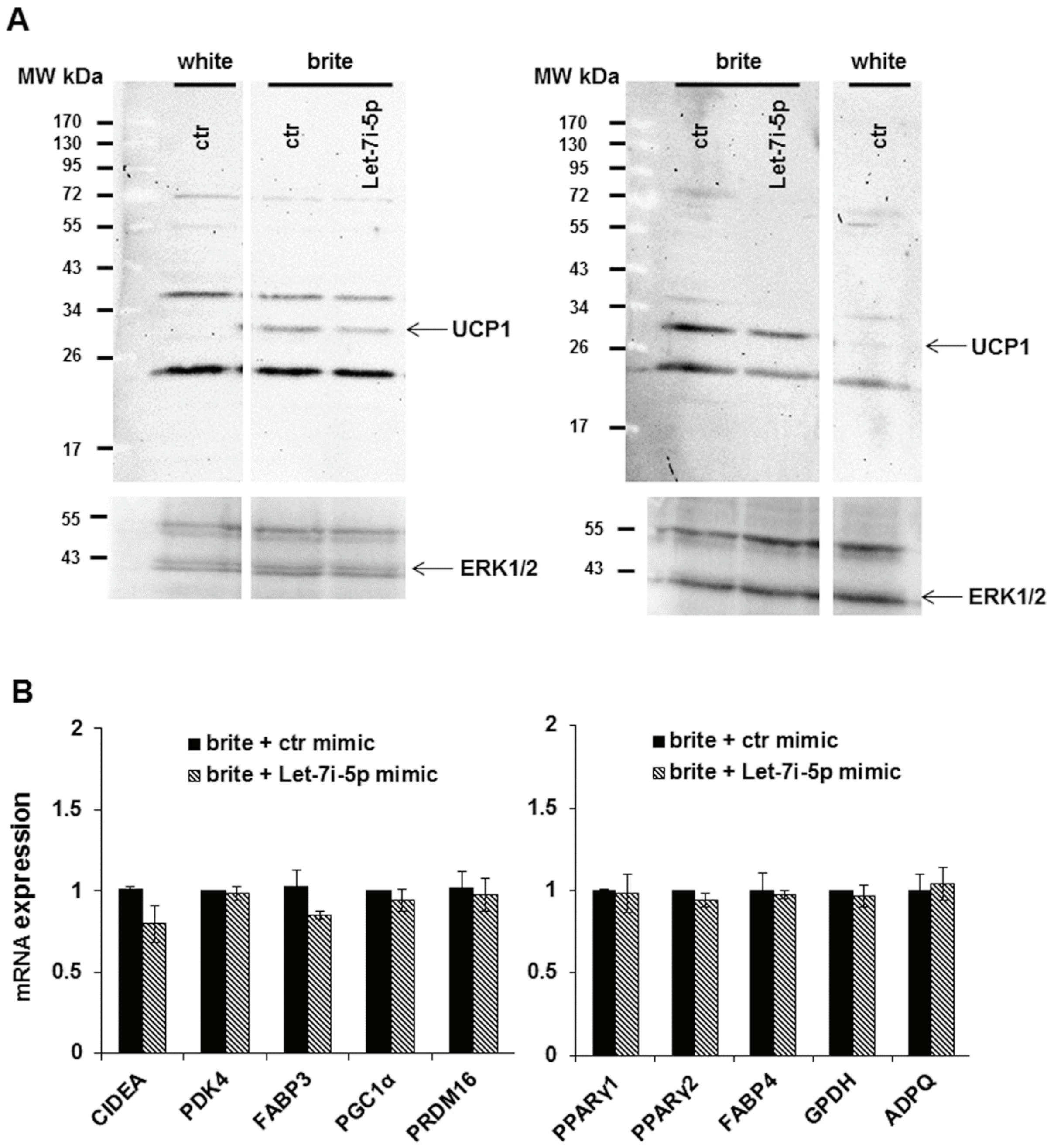

Supplemental Figure 2

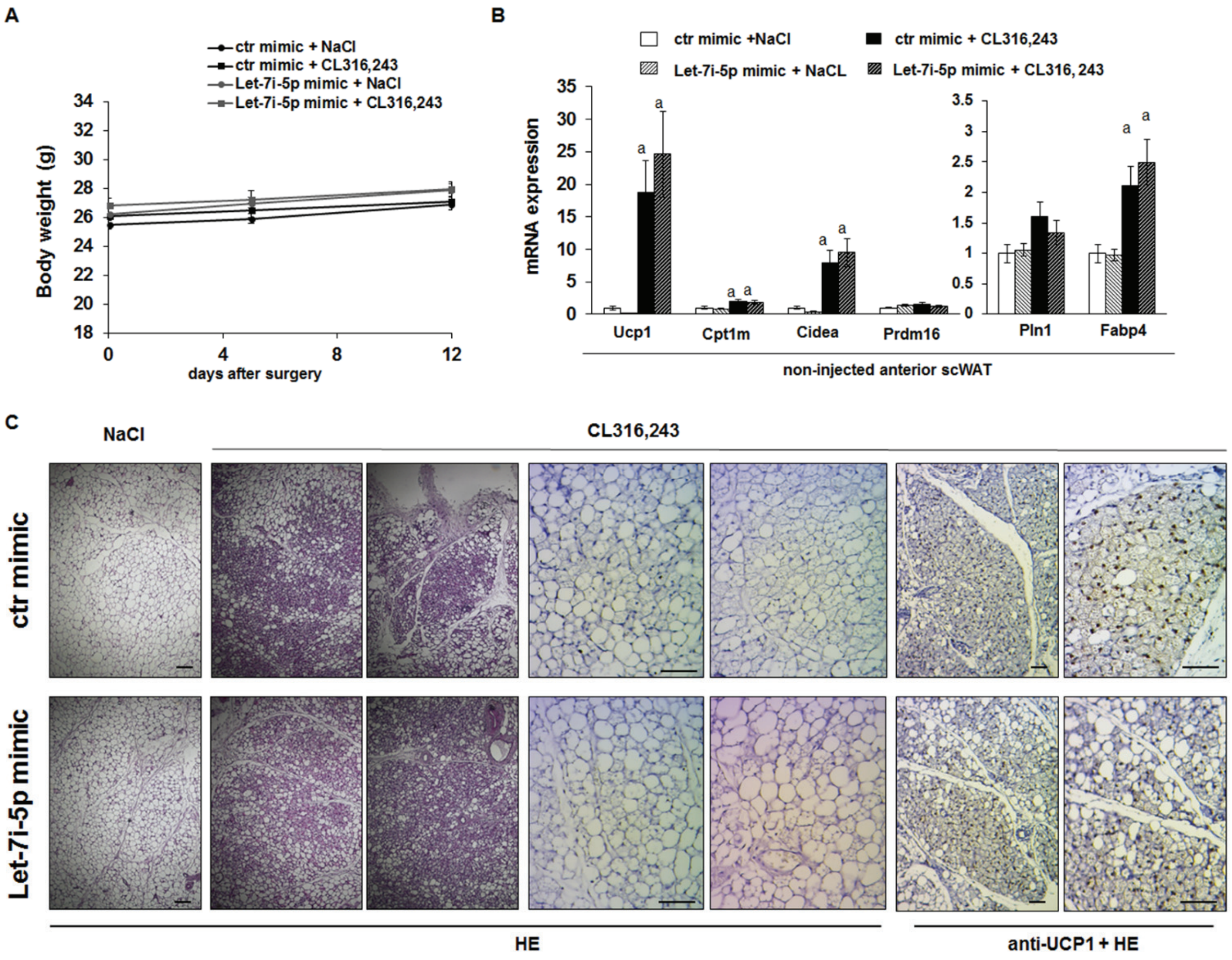

Supplement: Supplementary Information [file srep28613-s1.pdf]
